# Juvenile chronic non-bacterial osteomyelitis (CNO): Long term course of disease and response to treatment in a large institutional cohort

**DOI:** 10.1186/1546-0096-13-S1-P178

**Published:** 2015-09-28

**Authors:** T Schwarz, S Petzke, H Morbach, C Hofmann, M Beer, P Raab, HJ Girschick

**Affiliations:** 1 Northwest German Center of Rheumatology, St. Josef-Stift, Department of Pediatric Rheumatology, Sendenhorst, Germany; 2 University of Würzburg, Department of Pediatrics, Würzburg, Germany; 3 University of Ulm, Department for Diagnostic and Interventional Radiology, Ulm, Germany; 4 University of Würzburg, Department of Orthopaedic Surgery, Würzburg, Germany; 5 Vivantes Klinikum-Friedrichshain, Children's Hospital, Berlin, Germany

## Introduction

Chronic non-bacterial osteomyelitis (CNO) is an inflammatory disorder of the skeletal system of unknown etiology. Long-term follow-up and response to treatment data have rarely been reported. The aim of the study was to characterize the clinical, radiological, histological and laboratory data at juvenile CNO onset, and to analyze the long term treatment response.

## Methods

The course of disease of 93 juvenile patients (58% female) with non-bacterial inflammatory bone lesions was evaluated retrospectively. 1098 patient visits and 558 MRI findings were reviewed. Clinical, radiological, histological and laboratory data were assessed at disease onset and for a median time of disease of 46 months.

## Results

The mean age at disease onset was 10.9 years, the mean time between the first symptoms and the diagnosis of CNO was 11 months. 84% of the patients had multifocal bone lesions. Biopsy was performed in 80 patients. Only when bone biopsy was taken within 12 months of symptom onset, cellular infiltrates could be observed. At later time points, fibrosis, hyperostosis and bone edema predominated. 25% of all patients developed peripheral arthritis, 8% inflammatory bowel disease. The initial treatment consisted of non-steroidal anti-inflammatory drugs (NSAIDs). 37% of the patients required second line therapy consisting of sulfasalazine and short term oral corticosteroids, 8% of the patients required bisphosphonates or TNF-blocking agents. Median time to first clinical remission was 6.0 months and 94% of patients achieved clinical remission within the period under review. Median time to first radiological remission, however, was 27.4 months and only 67% of all patients achieved complete radiological remission. Time to remission was independent of the CNO being unifocal or multifocal. In detail analysis of the treatment response revealed that initiation of sulfasalazine treatment in NSAID non-responders led to a significant and sustained decline of the clinical, as well as the radiological number of lesions.

## Conclusion

The rapid clinical improvement in CNO, following initiation of therapy with NSAIDs, is not accompanied by a likewise decrease of the number of radiological lesions. Treatment with sulfasalazine is effective in childhood CNO.

